# Non-invasive muscle contraction assay to study rodent models of sarcopenia

**DOI:** 10.1186/1471-2474-12-246

**Published:** 2011-10-28

**Authors:** Chi-Sung Chiu, Hans Weber, Sharon Adamski, Albert Rauch, Michael A Gentile, Stephen E Alves, Gary Kath, Osvaldo Flores, Hilary A Wilkinson

**Affiliations:** 1 Department of Molecular Endocrinology, Merck Research Laboratories, West Point, Pennsylvania 19486, USA; 2 Research Operations, Merck Research Laboratories, West Point, Pennsylvania 19486, USA; 3 Research Operation, Merck Research Laboratories, Rahway, New Jersey 07065, USA

**Keywords:** fatigue, Boltzmann equation, dexamethasone, castration.

## Abstract

**Background:**

Age-related sarcopenia is a disease state of loss of muscle mass and strength that affects physical function and mobility leading to falls, fractures, and disability. The need for therapies to treat age-related sarcopenia has attracted intensive preclinical research. To facilitate the discovery of these therapies, we have developed a non-invasive rat muscle functional assay system to efficiently measure muscle force and evaluate the efficacy of drug candidates.

**Methods:**

The lower leg muscles of anesthetized rats are artificially stimulated with surface electrodes on the knee holders and the heel support, causing the lower leg muscles to push isometric pedals that are attached to force transducers. We developed a stimulation protocol to perform a fatigability test that reveals functional muscle parameters like maximal force, the rate of fatigue, fatigue-resistant force, as well as a fatigable muscle force index. The system is evaluated in a rat aging model and a rat glucocorticoid-induced muscle loss model

**Results:**

The aged rats were generally weaker than adult rats and showed a greater reduction in their fatigable force when compared to their fatigue-resistant force. Glucocorticoid treated rats mostly lost fatigable force and fatigued at a higher rate, indicating reduced force from glycolytic fibers with reduced energy reserves.

**Conclusions:**

The involuntary contraction assay is a reliable system to assess muscle function in rodents and can be applied in preclinical research, including age-related sarcopenia and other myopathy.

## Background

Age-related sarcopenia is associated with significant loss of muscle mass and muscle strength upon aging. The etiology of age-related sarcopenia is believed to be multi-factorial and includes aging, disease, inflammation, increased oxidative stress, reduced physical activity, malnutrition, hormone deficiencies, muscle structural changes, and motor unit remodeling [1]. By 2050, it is predicted that the worldwide population ≥ 60 years of age will more than triple from 600 million at present to 1.9 billion. This is likely to result in a growing need to treat aging related diseases such as age-related sarcopenia [2].

Although, there is no widely accepted clinical definition of age-related sarcopenia, several diagnostic protocols have been developed such as handgrip strength, knee extension isometric torque, lower extremity muscle power, weight loss, gait speed, physical activity questionnaires, dynamometer studies, and a standardized physical performance test battery of standing, walking, and stair climbing [3-10]. Generally, clinical tests for skeletal muscle function in sarcopenic patients are frequently performed on the lower extremities since the extremities show the most significant muscle degeneration during aging and because sarcopenia is frequently associated with reduced blood flow in these limbs [11,12].

Aged muscles show significant alterations to their architecture [13], underscored by a general loss of muscle fibers and a decrease in fiber size accompanied by motor unit remodeling. This remodeling predominantly affects fatigable glycolytic type II fibers that make the greatest contribution to strength [14]. Recently, several potential age-related sarcopenia targets and therapeutics have been identified and investigated, including androgen receptors, myostatin, TGF-β1 and Notch-1 signaling, multiple histone deacetylases, Angiotensin II pathway signaling, and β2-adrenoreceptor activators [15-19]. Some success in early phase clinical trials has been reported with ACE (Angiotensin converting enzyme) inhibitors [20] and selective androgen receptor modulators [21].

The animal models used in this report are dexamethasone (Dex) treated rats and naive aged rats. Dex is potent glucocorticoid and treatment of rats with Dex to induce myopathy is a frequently used model to evaluate drug candidates which affect muscle size or function [22,23]. Glucocorticoids, induce acute myopathy within a few days and reduce muscle force predominately due to atrophy of type II muscle fibers, without affecting specific muscle force [24]. While Dex-induced muscle atrophy selectively affects fast fibers, there are no gross changes in architecture which may explain the lack of alteration in specific force.

Aging rats start losing muscle mass in their lower extremities at ~18 months and become significantly weaker than adult rats of comparable size over a period of 4-6 months. Aging rats have many characteristics in common with humans regarding progressive changes in skeletal muscle architecture and selective loss of Type II alpha motor neurons and fast fibers with age [25,26]. As described above, therapeutic agents which affect muscle size in humans also increase muscle size and function in aging rats and mice [19,27-29].

Most preclinical approaches to evaluate potential therapeutics in animals have been focused on improving muscle structure and identifying biomarkers. However, the need for a reliable functional muscle assay that has sufficient throughput to be a practical tool in preclinical rodent studies has met with limited success. Preclinical animal studies to measure muscle function include grip strength, rotarod performance, treadmill performance, dynamometer performance, and in-vitro muscle functional assays of isolated muscle fibers.

The interpretation of results from most of the *in-vivo *assays is complicated by inherent variability and is limited by uncontrollable voluntary behavioral factors and in many cases, requires training and acclimation [30,31]. These factors can be especially difficult for older animals because both the reflexive response and the learning process in aged animals can be compromised compared to adult animals. *In-vitro *force measurements on electrically stimulated muscle fiber requires invasive fiber isolation procedures with lower throughput and are only applicable to study endpoints [32,33]. The rodent dynamometer evaluates isometric muscle force as well as force-velocity and force-power relationships under isokinetic and isotonic conditions [34-36]. A non-invasive hydraulic muscle assay system was developed to monitor muscle force using external electrode stimulation, a hydraulic piston system, and a pressure sensor [37]. The later two assay systems are conducted under anesthetic condition, hence eliminate voluntary factors during force measurement. Furthermore, the team of Giannesini et al stimulated muscle contraction using non-invasive external electrodes. The non-invasive external electrode stimulation enables the performance of multiple studies on the same animals; it induces contractions of more than one muscle group so that it allows evaluation of muscle strength resulting from the combination of these forces. It is not suitable for characterization of individual muscle contraction. The involuntary assay systems are not commercially available and the throughput remains low. We aimed to develop a higher throughput involuntary contraction assay system that enables routine in-vivo studies to screen compound efficacy to support drug discovery.

We developed the rodent involuntary muscle contraction system that electrically stimulates the lower hind limb muscles of anesthetized rats and measures the isometric force output by placing force transducers behind the foot pedals. This system eliminates animal voluntary factors and measures muscle functionality.

By using the custom built animal assay stage and the automated data analysis, this system has been optimized to have reasonable throughput for supporting routine animal studies in drug discovery. We wanted to determine whether this system was robust and sensitive enough to detect muscle performance changes in two different preclinical models of muscle loss. If this system met these criteria then it would be widely applicable for discovery of therapeutics to treat diseases and conditions of muscle loss.

## Methods

### Animals and Animal Models

All animals were housed under equal day and night cycles and were fed a standard diet *ad libitum*. All animal procedures were approved by the Merck Research Laboratories-West Point, PA IACUC in accordance with the National Research Council Guide for the Care and Use of Laboratory Animals.

#### Aged and adult rat model

Aged and adult retired male breeder Sprague Dawley rats at 24 months old (Charles River, Wilmington, MA) and 9 to 11 months old (Taconic, Hudson, NY) respectively were examined. The expected life span of these rats is approximately 30 months. They start to show mortality by 18 months of age and show higher mortality rates and significant muscle loss by ~24 month of age.

The body composition of the adult retired breeder rats was determined by quantitative NMR (qNMR) and results were very consistent from animal to animal. The body compositions of 315 aged rats were also determined by qNMR and there was a high degree of variability among the animals so they were sub-grouped into heavy, light, lean, and fat body type groups. Six individual rats were allocated to each group based on body weight, lean mass, fat mass, fat/lean ratio, and fat/body weight ratio. The aged heavy and aged light animals were differentiated based on their body weight being significantly greater or less, respectively, than the average of the aged rats and the adult rats (average body weight for adult, aged heavy and aged light animals was 593 ± 14 g, 766 ± 11 g and 574 ± 17 g, respectively). The aged fat animals were differentiated based on having a significantly different distribution of lean and fat (the fat/lean ratio was 21% for adult rats, 21% for aged lean rats, 42% for aged heavy, 40% for aged light and 55% for aged fat). The lean animals were differentiated based on having a similar weight and body composition to the adult rats.

#### Dexamethasone treated rat model

Male retired breeder Sprague-Dawley rats (10 - 12 month old, ~550 g) (Taconic, Hudson, NY) were implanted with 90 day delayed release pellets containing either 1.5 mg dexamethasone to achieve a dose of 0.03 mg/day or placebo pellet (Innovative Research of America, Sarasota, FL). Rats were tested 11 days after pellet implantation, since this was the time period when the most profound muscle atrophy was observed.

### Body Composition Measurements

In live animals, fat mass and lean mass were measured using the EchoMRI 700**™ **following the manufacturer's guidelines (Echo Medical Systems, Houston, TX). Animals are weighed on a scale to determine total body mass and then subjected to necropsy, by CO2 euthanasia, at the end of each study. The plantarflexor muscle group was dissected from both legs and the right side muscles are weighed. A post hoc analysis using the Tukey-Kramer test was used for multiple group comparisons.

### Involuntary Contraction Assay

**Figure 1:**
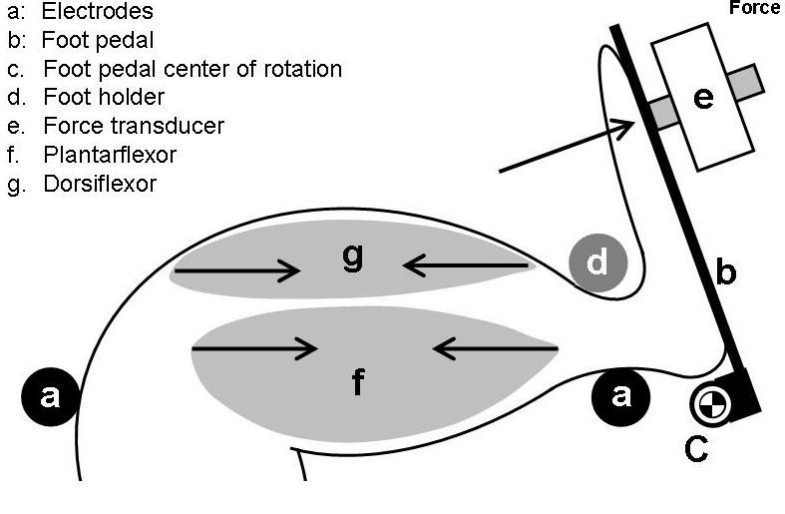
**Diagram of rodent hind limb involuntary contraction assay**. The system includes surface electrodes placed at the knee and heel (a), a foot pedal (b), and a force transducer (e). Contraction of the plantarflexor muscles (f) exerts force onto the transducer (arrow).

**Figure 2:**
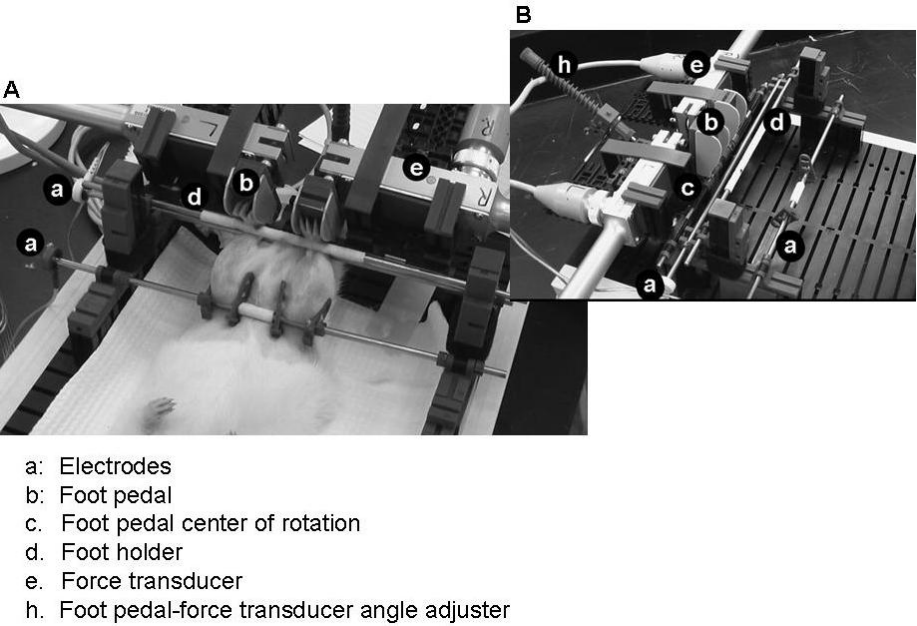
**Images of the rat involuntary contraction assay stage**. The electrodes (a), foot pedal (b), foot holder (d) and foot pedal center of rotation (c), force transducer (e) and foot pedal-force transducer assembly (h) are labeled on the images.

The involuntary contraction assay stage was built using building blocks from a construction set (Fischertechnik **^®^**, Germany) on top of a base plate. It also includes a gas anesthesia nose cone, two 1/8" stainless steel rod electrode (one at the knee and one at the heel), one 1/8" stainless steel rod foot holder, two foot pedals with hinge on the base to a stainless rod, and two force transducers (FT10, Grass Instruments, West Warwick, RI) that are attached to the foot pedals. The rats are anesthetized with isoflurane (3-5%) and are placed belly up on the stage. Hind paws are placed on the pedals with the femur bone roughly perpendicular to the tibia bone (Figure 1; Figure 2A and Figure 2B). The hind limb is secured tightly against the foot pedal by the electrode rods on the knee and beneath the heel and a stainless rod on the top of the ankle. The angle of the pedal-force transducer assembly is adjustable to allow for a natural position (usually ~20o backward) of the ankle joint. The placement and level of stimulation of the external electrodes on the knee and heel was optimized to result in generation of maximum observable force. This stimulation results in movement of the foot on the foot pedal which presses against the force transducer.

Stimulation waveforms were generated by an analog constant voltage stimulator (S48, Grass Instruments, West Warwick, RI) and a 10 Volt signal was split and sent two DS3 constant current isolated stimulators (Digitimer, Hertfordshire, England), where the amplitude of the stimulation was set in mA. Each DS3 stimulates one leg. Force transducer signals were amplified (P122, Grass Instruments, Warwick, RI), digitized and recorded at 1 kHz (PolyView 16, Grass Instruments, Warwick, RI).

### Fatigability Test

**Figure 3:**
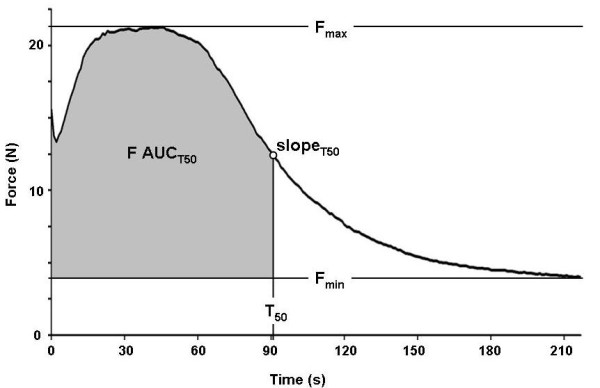
**A typical fatigue envelope recorded from a rat includes a force potentiation phase, followed by a plateau at maximum force, a fatiguing phase, and a slow-fatiguing or fatigue-resistant phase**. Parameters of a fatigue envelope include maximum force (Fmax), minimum force (Fmin), half fatigue time (T50), slope at T50 (slopeT50), and a fatigable muscle force index (F AUCT50).

To simulate a fatiguing exercise, tetanic contractions were evoked repeatedly by sending supramaximal square wave pulse trains to the leg muscles. Stimulation parameters included pulse duration, pulse frequency, train duration, train frequency, and stimulation amplitude. Each tetanic contraction generates a force spike. The maximum force of each spike was obtained from the recorded raw data using a custom LabView **^® ^**script (National Instruments Corporation, Austin, TX) and exported as a fatigue envelope (Figure 3) for graphing and further analysis, such as curve fitting. A two-way ANOVA (force, time) and Bonferonni's post hoc test were used to compare differences between groups in force during fatiguing stimulation.

### Fatigue envelope analysis

Natural muscle aging and glucocorticoid treatment both result in a preferential loss of type II fibers [38,39]. Curve analysis was applied to approximate the contributions of fatiguing fibers (fast fatigable muscle myofibers, type II fibers) and fatigue-resistant fibers (slow-/non-fatigable myofibers, type I fibers) to the total force output. The fatigue envelope was curve fitted with a Boltzmann equation that generated four parameters: maximum force (Fmax), minimum force (Fmin), half fatigue time (time at (Fmax-Fmin)/2, T50), and fatigue slope (slope of the fatigue envelope at half fatigue time, SlopeT50) [40]. Fmin was selected to represent fatigue resistant muscle force and it was not observed to change significantly during the recording (Figure 3) [38]. The Fmax and Fmin are defined as the highest and the lowest muscle forces that are generated under a specific stimulation protocol. To approximate the contribution of fatigable muscle force, we took Fmax force values before T50 subtracted Fmin and integrated the area under the curve to represent a fatigable muscle force index (F AUCT50; Figure 3).

The indices can be used as a ratio for comparison between different groups in the same study. One-way analysis of variance (ANOVA) (α = 0.05 or 0.01) was used to determine differences between the tested groups.

## Results

### Fatigability Test Development

**Figure 4:**
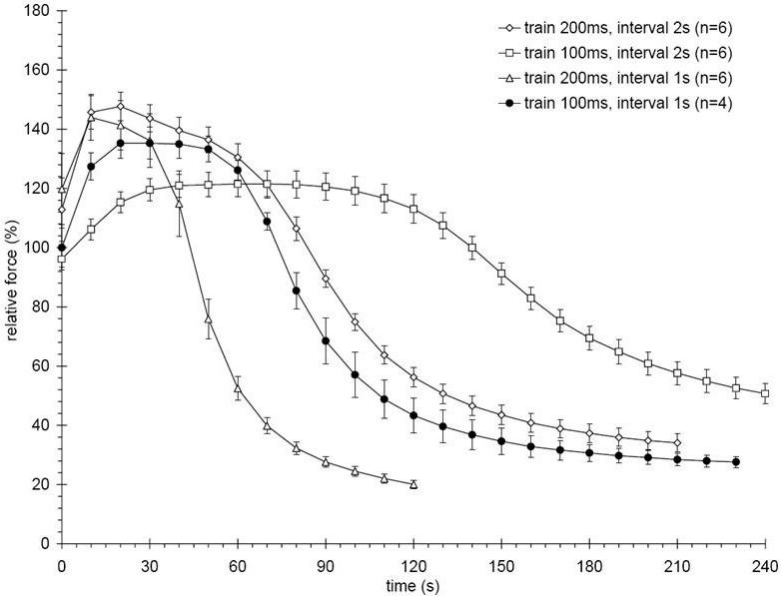
**Fatigue envelopes for different stimulation protocols**. Hind limb muscles are stimulated with trains of either 100 or 200 ms at either 1 or 2 s intervals. Each stimulation protocol generates different peak forces, plateau force durations, and fatiguing slopes. Both 200 ms protocols showed the highest maximum force, but no sustainable plateau. The 100 ms at 1 s interval protocol was chosen, because it showed a sustained force plateau and fatigued the muscles in less than 4 minutes. Error bars are mean ± SEM.

To optimize the stimulation protocol for tetanic force development, we compared single tetanic stimulations with different pulse duration and found that pulse duration of 1 ms at a frequency of 60 Hz and an amplitude approximately 1.5 times the maximal effective stimulation amplitude, resulted in a reliable supramaximal stimulation and a fully fused tetanus (data not shown). To optimize the fatigue related stimulation parameters, we tested four different protocols on ~10-12 month old rats and generated fatigue envelopes (Figure 4).

The chosen protocol used a train duration of 100 ms at a train frequency of 1 Hz and produced the most suitable fatigue envelope which contained a stable plateau force at around the maximum force for over 30 s and reached a fatigue-resistant state within 2 min. The other protocols either needed a longer time to reach fatigue resistance or were too demanding to represent everyday muscle use as indicated by the lack of a plateau at maximum force.

### Muscle force changes differently among aged rats with different body composition

**Figure 5:**
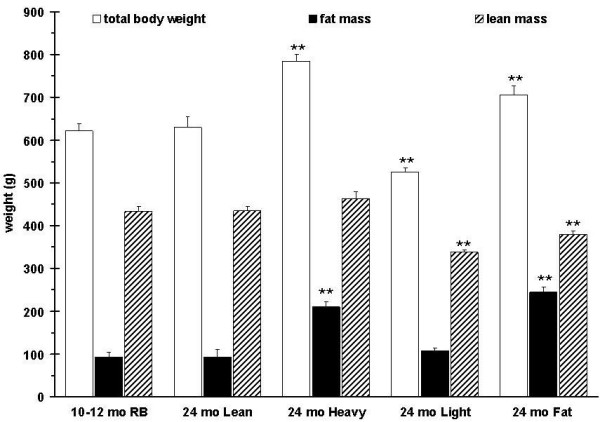
**Body composition of adult retired breeder rats (10-12 month old retired breeders (RB)) and 4 subgroups of aged rats (24 months)**. The subgroups (n = 6 for each subgroup) were screened from 315 aged rats based on body composition parameters (fat mass, lean mass, body weight (BW)). The lean subgroups body composition is indistinguishable from adult rats. P values are given compared to 10-12 month old RB.

**Figure 6:**
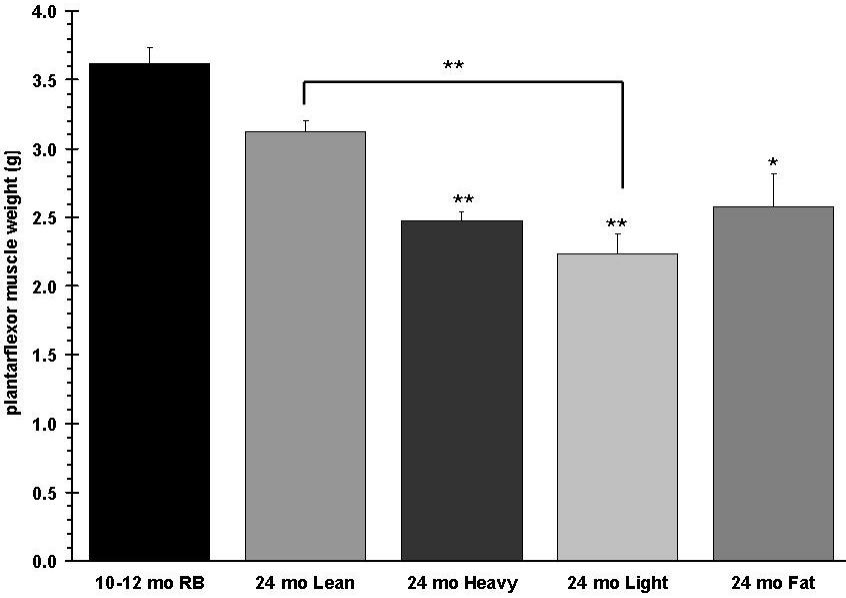
**Plantarflexor (gastrocnemius, plantaris, soleus) muscle group weights are shown**. P values are given compared to 10-12 month old RB.

Body weights and qNMR scans of 315 rats at 24 months of age revealed a number of body composition types. We assembled four representative groups, i.e. heavy, light, fat, and lean, to compare with adult10-12 month old rats (Figure 5). The aged lean group showed no significant differences in body composition from the 10-12 month old rats. The weight of the plantarflexor muscle was heavier in the adult rats when compared to all aged rat subgroups. The lean subgroup had the next heaviest muscle followed by the fat subgroup (Figure 6). If the plantarflexor muscle mass is adjusted relative to the body mass then the aged rat groups are not different from each other but they are significantly different from the adult rats (relative plantarflexor mass is 0.0057 g ± 0.0001 g SEM for adult, 0.0050 g ± 0.0001 g SEM for aged lean, 0.0032 ± 0.0003 g SEM for aged heavy, 0.0044 ± 0.000 g SEM for aged light, and 0.0036 ± 0.0002 g SEM for aged fat groups).

**Figure 7:**
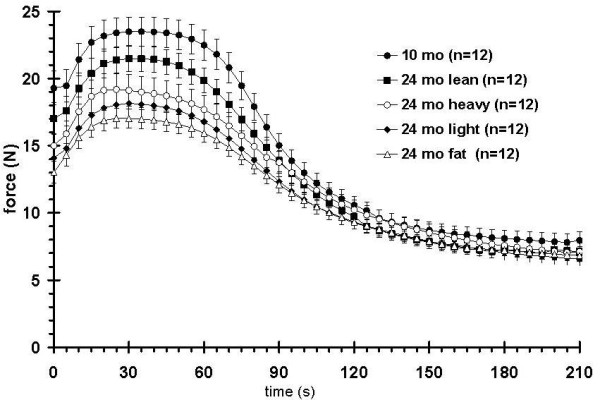
**Fatigue envelopes of adult retired breeders (RB) and aged rats of different body composition**. The adult rats show the strongest muscle force while the lean subgroup of the aged rats ranks second. The other aged subgroups are overall weaker but not significantly different from each other.

**Figure 8:**
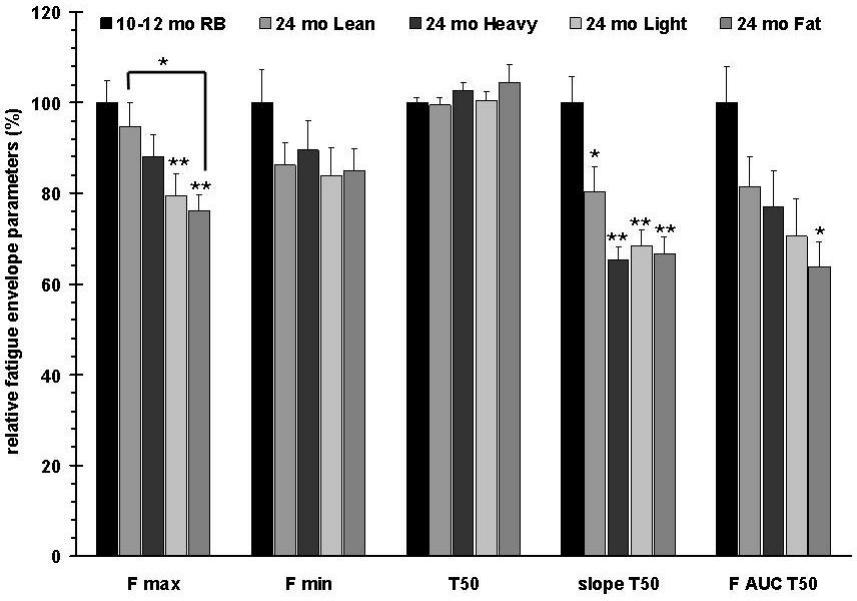
**Calculated fatigue envelope parameters**. Compared to the 10-12 months old retired breeders (RB), the aged subgroups show a graded Fmax reduction, while reductions of slope T50 and F AUC T50 are more pronounced. No differences were observed between adult rats and all aged rat subgroups for T50. Error bars indicate mean ± SEM. P values are given compared to 10-12 month old RB.

The groups were also tested with the involuntary contraction assay (train duration of 100 ms at a train frequency of 1 Hz) (Figure 7), in which the adult rats had the strongest muscle force, followed by the aged lean group. Using two-way ANOVA analysis (Bonferroni posttests) on the muscle forces during the first 90 s of contractions the muscle forces generated by adult rats are significantly stronger than all aged groups, except the aged-lean group. Among the aged groups, only the aged-lean group showed significantly stronger force than the aged-fat group between 20 s and 55 s after the beginning of the stimulation. The heavy, light, and fat groups exhibited weaker force with no significant differences among the three groups. With the exception of the aged fat subgroup, the performance of all groups corresponded to the mass of their hind limb plantarflexor muscles (Figure 6). To approximate the forces that were contributed from the fatigable and fatigue-resistant myofibers, fatigue envelopes were curve fitted and half fatigue times as well as fatigue slopes were plotted. All aged rat subgroups showed a significantly reduced Fmax and slope T_50 _compared to the adult rats. F AUCT50 was significantly lower in the aged fat group compared to the adult rats, but there was no significant difference in F AUCT50 between the other aged subgroups and adult rats. Fmin and T_50 _were not different between groups (Figure 8). ANOVA analysis of muscle force from all subgroups at the first 90 s of the stimulation indicated that adult rats displayed significantly stronger force compared to the heavy, light, and fat aged subgroups, however no significant difference was found between the adult rats and the aged lean subgroup. On the other hand, the aged lean subgroup had significantly higher force values than the aged fat subgroup (data not shown).

### Muscle force is decreased in a rat model of dexamethasone induced myopathy

**Figure 9:**
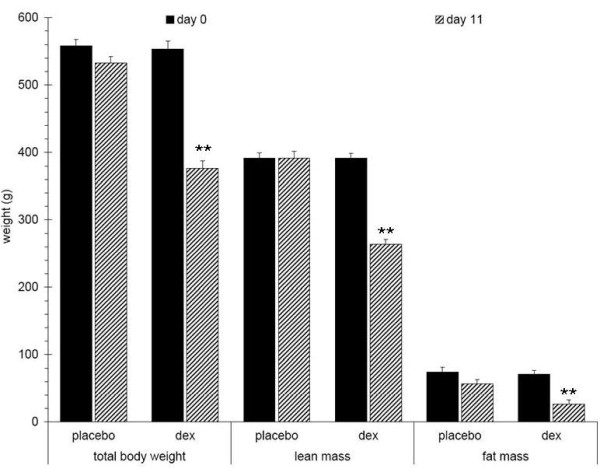
**Changes in body composition of Dex treated 10-12 month old male rats (n = 12)**. Dex treated rats show significantly decreased body weight (30%), lean mass (33%), and fat mass (53%) after 11 days when compared to placebo treated rats. P values are given compared to placebo treated rats.

**Figure 10:**
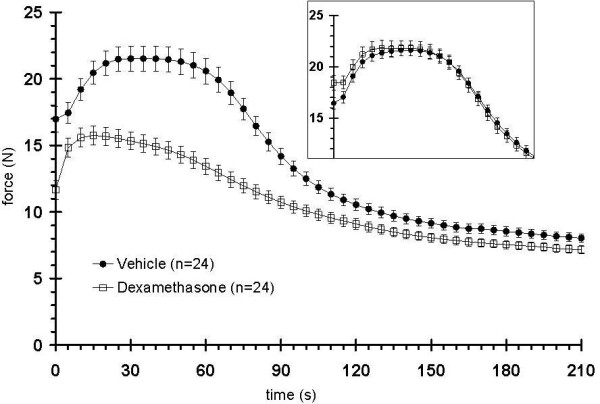
**Changes in fatigue envelopes of Dex treated 10-12 month old male rats (n = 12)**. The fatigability test, performed before treating with Dex showed no differences in muscle force (inset). After 11 days of treatment, the Dex treated rats showed a significantly reduced force plateau (fatigue envelopes from both legs were recorded). Twelve rats from each treatment group were tested and the force generation of their left and right legs were recorded independently (i.e. n = 24 data sets/group).

**Figure 11:**
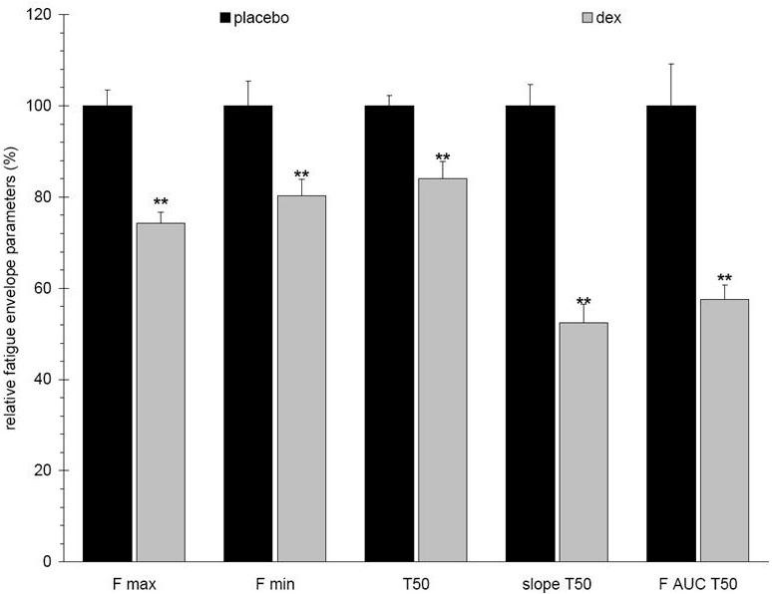
**Changes in fatigue envelope parameters of Dex treated 10-12 month old male rats (n = 12)**. Dex treated rats show reductions in all fatigue envelope parameters, e.g. 25%, 20%, 15%, 50%, and 45% reductions of Fmax, Fmin, half fatigue time (T50), slopeT50, and F AUCT50, respectively. Error bars indicate mean ± SEM. P values are given compared to placebo treated rats.

Over two weeks of Dex treatment, rats exhibited a time dependent decrease in total body mass, lean mass, and fat mass, with a loss of hind limb muscle mass proportional to the overall loss of lean mass (data not shown). In this study, after 11 days of Dex treatment, animals had reductions in total body mass, lean mass, and fat mass of about 30, 33, and 53% respectively, when compared to placebo controls (Figure 9). The plantarflexor muscles were also reduced by 30% as compared with matching controls (data not shown). Fatigue envelopes showed similar profiles at baseline (Figure 10 inset); at study end however, Dex treated rats showed a significantly reduced fatigable muscle force, consistent with a loss of type II fibers (Figure 10; Menezes 2007, Seene 2003). Interestingly, Dex treated rats reached the curve center at 50% fatigue which is significantly faster than control animals (74 vs. 87 contractions) and they did not show a typical Fmax plateau around maximal force but instead started fatiguing right after reaching maximum force. The Dex treated rats showed reduced muscle performance in all parameters and the slopeT50 and F AUCT50 were the most affected parameters (Figure 11).

## Discussion

The described non-invasive involuntary muscle contraction assay allows repeated monitoring of skeletal muscle isometric force during preclinical drug efficacy studies in rodents with throughputs of up to 8 animals per hour. This system measures muscle force on anesthetized animals, which eliminates the voluntary behavior factor and enables measurement of the functional output of the leg muscles. This assay system is designed to repetitively and non-invasively monitor rat muscle functionality in response to drug treatment. This non-invasive assay system allows repetitive monitoring of muscle functionality in the same animal at multiple time points during a long term study.

There are limitations to the interpretation of changes in muscle force data from this type of evaluation. Although the hind limb muscles were stimulated by supra-maximum current, it is unknown whether both the plantarflexor and the dorsiflexor muscle were fully activated; this assay measures the sum of forces contributed by the agonist and antagonist effects of the plantarflexor and dorsiflexor muscle groups. The system provides an indirect measurement of muscle force generated by contractions of multiple muscles, and is not designed to assess the force contributions from specific muscle groups or fiber types. It is expected that some of the actual muscle force is dissipated by mechanical friction during transmission to the force transducer and by the paw/pedal acting as a lever. Nevertheless, the fatigue envelop generated from the involuntary contraction assay shares a similar profile with isolated muscle tested in an organ bath [41,42]. Still, the parameters calculated from the fatigue envelopes are relative indicators of the phenotype rather than documenting the true changes of muscle force.

Using the involuntary contraction assay to perform muscle fatigue tests provided well defined fatigue envelopes that show phases of force potentiation, Fmax plateau, fatiguing, and fatigue resistance, thus providing information about the amount of fatigable muscle force, its rate of fatiguing, and the amount of remaining fatigue-resistant force for each subject [38]. Similar fatigue envelopes are frequently used in clinical diagnosis of muscular diseases and injuries, as well as in a wide array of basic research studies on isolated mammalian skeletal muscles [38,42-45]. Most importantly, this approach promises to bridge the gap between classical preclinical muscle studies and current clinical research [43,45,46].

The fatigue slope (Slope T50) and the fatigable area under the curve (F AUCT50) are useful parameters to describe muscle functionality in this test. The calculation of functional indices of approximate force contributions of fatigable and fatigue-resistant muscle fibers may enable us to distinguish functional drug effects on myofiber subtypes. However, using Fmin as a cutoff to approximate the contribution of fatigue-resistant muscle fibers to the total force before the curve center is likely to result in an underestimate, because those fibers do show some fatiguing during the latter part of the muscle fatiguing test. Therefore the fatigable index might also be higher than estimated. In the Dex study, this effect might skew the comparison of the functional index ratios between vehicle and Dex treated groups, since Fmin is very similar in both groups and therefore relatively larger in the weaker Dex treated rats. Nonetheless, comparing functional indices and their ratios within a study should provide relevant information about differences in fiber type composition of the tested leg muscles.

Further investigation including fiber type histology will be necessary to refine our approximations. Most aged rats showed a profound loss of fatigable force and a somewhat diminished fatigue-resistant force when compared to their adult counterparts, as indicated by comparing Fmin and F AUCT50. It has been reported in aging rats that the stronger type II myofibers degenerate faster than the fatigue-resistant type I [39] and our results are consistent with these observations, however immunohistochemical characterization of the muscle is needed to confirm the distribution of fiber types. The lean group showed the best performance among the aged subgroups, while the significantly weaker muscle function of the fat group could be due to a higher infiltration of fat within the muscle architecture [47]. Dex treated animals showed a significant reduction in fatigable muscle force but only a relatively minor reduction of fatigue-resistant force, which confirms previously published results where type II fibers were significantly more affected by Dex treatment than type I [22,48] and provides support for Dex treatment as a potential animal model to represent muscle aging. The lack of an Fmax plateau and a shorter half fatigue time (T50) could be due to reduced energy resources for type II fibers, which would agree with reports indicating decreased glucose uptake and decreased glycogen synthesis in Dex treated rats [49].

## Conclusions

In summary, the fatigue tests that were performed in the two different studies, using our custom built involuntary contraction assay system, provided data that are validated by the literature. This indicates that the assay system is a reliable way to assess muscle function in rat. Overall, performing fatigue tests with the involuntary contraction assay has effectively revealed changes in functional muscle parameters in preclinical rodent studies and it promises to be a useful tool to evaluate the efficacy of sarcopenia treatments and may enhance drug discovery for muscular dystrophies as well. The described approach could be a reliable bridge between preclinical and clinical studies, since it should be translatable to clinical research.

## Abbreviations

(Dex): dexamethasone; (qNMR): quantitative NMR; (Fmax): minimum force; (Fmin): maximum force; (ANOVA): one-way analysis of variance; (Slope T50): fatigue slope; (F AUCT50): fatigable area under the curve; (T50): half fatigue time;

## Competing interests

All authors are or were employed by Merck and Company, Inc. at the time the paper was written. Dr. Osvaldo Flores has since left the company. The authors potentially hold stock or stock options in the company.

## Authors' contributions

CCS designed and coordinated in-vivo studies, animal necropsy, analyzed data, and drafted and revised the manuscript. WH built the involuntary assay stage, conducted the involuntary contraction assay, and revised the manuscript. AdS involved in animal dosing and body composition analysis and performed necropsy and tissue collection. RA involved in the assay stage mechanical and electrical design and involuntary contraction assay data acquisition and analysis.

GM involved in animal dosing and animal necropsy. AlS involved in drafting the manuscript and provided critical intellectual inputs. KG supported and commented the mechanical design of the assay stage and provided critical intellectual input. FO approved this study and provided critical intellectual input.

WHA approved this study, drafting and revising the manuscript, provided critical intellectual comments, and is the corresponding author. All authors read and approved the final manuscript.

## Pre-publication history

The pre-publication history for this paper can be accessed here:

http://www.biomedcentral.com/1471-2474/12/246/prepub
